# Health assessment of *Conolophus subcristatus*, *Conolophus pallidus*, and *C. subcristatus X Amblyrhynchus cristatus* hybrid (Galápagos land iguanas)

**DOI:** 10.1371/journal.pone.0222884

**Published:** 2019-10-16

**Authors:** Gregory A. Lewbart, Colon J. Grijalva, Paul P. Calle, Karen Ingerman, Juan Pablo Muñoz-Pérez, Galo Quezada, Carlos A. Vera, Gabriele Gentile, Carlos A. Valle

**Affiliations:** 1 Universidad San Francisco de Quito USFQ, Colegio de Ciencias Biológicas y Ambientales, Casilla, Quito, Ecuador; 2 Galápagos Science Center GSC, Av. Alsacio Northia, Isla San Cristobal, Galápagos, Ecuador; 3 North Carolina State University College of Veterinary Medicine, William Moore Drive, Raleigh, NC, United States of America; 4 Universidad San Francisco de Quito, Escuela de Medicina Veterinaria, Diego de Robles s/n y Pampite, Quito, Ecuador; 5 Wildlife Conservation Society, Zoological Health Program, New York, New York, United States of America; 6 University of the Sunshine Coast, Sippy Downs, Queensland, Australia; 7 Fundación Equilibrio Azul, Machalilla, Ecuador; 8 Dirección Parque Nacional Galápagos, Technical Biodiversity Research, Puerto Ayora, Galápagos, Ecuador; 9 Department of Biology, Tor Vergata University, Via della Ricerca Scientifica, Rome, Italy; University of Georgia, UNITED STATES

## Abstract

The land iguanas, *Conolophus pallidus* and *Conolophus subcristatu* are large and charismatic lizards endemic to the Galápagos archipelago, but little information exists on their normal health parameters. The former is restricted to Santa Fe island, while *C*. *subcristatus* inhabits the islands of the central and western region of the archipelago. Both species are classified as vulnerable by the IUCN Red List of Threatened Species. As part of a population health assessment authorized by the Galápagos National Park, wild adult iguanas from three islands (North Seymour, South Plazas, and Santa Fe) were captured in July 2018. Data from a single *C*. *subcristatus X Amblyrhynchus cristatus* hybrid captured on South Plazas is also included. We analyzed blood samples drawn from 52 healthy wild adult land iguanas captured on three islands. An iSTAT portable blood analyzer was used to obtain values for pH, lactate, pO2, pCO2, HCO3-, sO2%, hematocrit, packed cell volume (PCV), hemoglobin Na, K, iCa, and glucose. Standard laboratory hematology techniques were employed for PCV determination; resulting values were also compared to the hematocrit values generated by the iSTAT. Body temperature, heart rate, respiratory rate, and body measurements were also recorded and compared to previously published data for the marine iguana (*Amblyrhynchus cristatus*), which shares a common ancestor with the land iguana. The data reported here provide preliminary baseline values that may be useful in comparisons between captive and wild populations, between wild populations, and in detecting changes in health status among Galápagos land iguanas affected by anthropogenic threats, climate change, or natural disturbances.

## Introduction

*Conolophus pallidus* and *C*. *subcristatus* are land iguanas endemic to the Galápagos archipelago. The former species is restricted to Santa Fe island, while *C*. *subcristatus* inhabits the islands of the central and western region of the archipelago [1,2]. Both species are under the protection of Galápagos National Park (GNP) and classified as vulnerable by the IUCN Red List of Threatened Species [3,4]. As part of a population health assessment authorized by the GNP, wild iguanas from three islands (North Seymour, South Plazas, and Santa Fe) were captured in July 2018. Veterinary health examinations, that included sampling blood, ectoparasites, and feces, were performed on each animal in accordance with the ethics and animal handling protocols of GNP. Physical examination included measurement of body temperature, heart rate, length, and weight.

Peripheral blood biochemical, blood gas, and hematology parameters are useful for assessing lizard health [5,6]. Since factors such as disease, injury, pollutants, or starvation can cause perturbations in blood values, it is important to establish species-specific baseline reference range parameters for healthy individuals and populations. Reference intervals for certain iguanid species have been investigated. These include *Amblyrhynchus cristatus* [7], *Basiliscus plumifrons* [8], *Cyclura cychlura inornata* [9], *Cyclura ricordii* [10], and *Iguana iguana* [11–15]. In this study, hematology, blood biochemical, and blood gas values are evaluated for 52 healthy wild adult Galápagos land iguanas.

## Materials and methods

### Ethics statement

This population health assessment was conducted on three islands in the Galápagos archipelago and was authorized by the Galápagos National Park Service (Permit # PC-70-18 to G.A. Lewbart) and approved by the Universidad San Francisco de Quito ethics and animal handling protocol.

### Iguana capture, measurements, and sampling

A total of 52 healthy wild adult land iguanas (30 *C*. *subcristatus*, 21 *C*. *pallidus*, and one *C*. *subcristatus A*. *cristatus* hybrid) were sampled from three locations in the Galápagos Islands. Iguanas were captured by hand among brown earth and scrub foliage terrain within 200 m of the shore. The animals were quickly transported to the field laboratory (usually located within 100 meters of the capture site), examined, measured, weighed, and sampled. The cloacal temperature, heart rate, and respiratory rate were recorded as soon as the animal arrived at the field laboratory. This was usually within 3 minutes. Respiratory rates were measured by visualization and heart rate via a Doppler ultrasound probe (Parks Medical Electronics, Inc., Aloha, Oregon, USA) over the heart.

An EBRO^®^ Compact J/K/T/E thermocouple thermometer (model EW-91219-40; Cole-Parmer, Vernon Hills, Illinois, USA 60061) with a T PVC epoxy-tipped 24 GA probe was used to determine core body temperature. Axillary girth (AG), snout-vent length (SVL), and total length (TL) were recorded using an appropriate length measuring tape. Body weight was measured with a digital scale (precision: 0.1 kg). The sex of the iguanas could not be determined with confidence despite taking hemipenal sac depths with a stainless steel sexing probe.

Handling time, the average interval between iguana capture and blood sample collection, was 11.5 minutes for *C*. *subcristatus* and 3.95 minutes for *C*. *pallidus*. Twenty *C*. *subcristatus* were captured, examined, and sampled from North Seymour Island (0^0^ 55’ 40” S, 89^0^ 36’ 43” W) on 25–26 July, 2018; 10 *C*. *subcristatus* and one *C*. *subcristatus X A*. *cristatus hybrid* were similarly captured and sampled at South Plazas Island (0^0^ 34’ 55” S, 90^0^ 9’ 59” W) on 26–27 July, 2018. Finally, 21 *C*. *pallidus* were captured, examined, and sampled from Santa Fe Island (0^0^ 48’ 04” S, 90^0^ 2’ 45” W) on 28 July, 2018. Before release, ectoparasite load was quickly inspected by counting the number of ticks (*C*. *subcristatus*) or by recording their presence / absence when infestation was by a large number of very small ticks in the case of *C*. *pallidus*. To avoid capturing the same individual more than once, a line of white zinc oxide ointment, approximately 10 X 2 cm in size, was applied to each animal’s dorsal trunk before the lizard was released. Each animal was scanned to determine if it had a microchip present from previous examinations, and if it did not, a microchip was placed intramuscularly. In cases where ticks were found several were removed and preserved in 70% ethanol.

### Blood sampling and handling

A blood sample of approximately 2.5 mLs was obtained from the coccygeal hemal arch of each manually restrained iguana using a heparinized 22 gauge 1.0 in. needle attached to a 3.0 mL syringe. The blood was divided into sub-samples; several drops were used for making blood films on clean glass microscope slides, one drop was used for lactate analysis, about 0.1mL was loaded into the Chem8 iSTAT cartridges, and about 0.05 mL was used for centrifugation with a portable microcentrifuge (Eppendorf North America, Inc., centrifuge model 5424, 5 min. at 14,000 G) to determine PCV and total protein. The PCV was determined by measuring the percentage of cellular material compared to plasma in the tubes. Two drops of plasma were placed on a refractometer (Ade Advanced Optics, Oregon City, Oregon 97045, USA) and the values recorded. The remainder of the sample was stored on ice in sterile plastic vials in the field and then transferred to a refrigerator upon arrival in the laboratory for future analysis of the plasma.

### Biochemistry, blood gas, and hematology parameters

An iSTAT Clinical Analyzer (Heska Corporation, Fort Collins, Colorado, USA) and CG8+ cartridges were used to obtain biochemistry, blood gas, and electrolyte values. The iSTAT is a handheld, battery-powered, device that measures selected blood gas, biochemical, and hematology parameters using just 0.095 mL of non-coagulated whole blood. The following parameters were measured: Base excess in the extracellular fluid compartment (BEecf), glucose, bicarbonate (HCO_3_^-^), hematocrit, hemoglobin, ionized calcium (iCa), potassium (K), sodium (Na), partial pressure of carbon dioxide (pCO_2_), pH, partial pressure of oxygen (pO_2_)_,_ and percent oxygen saturation (sO_2_%). Temperature corrected values are automatically produced by the iSTAT for pCO_2,_ pH and pO_2_ once the animal’s body temperature is entered. Blood lactate was determined using a portable Lactate Plus^TM^ analyzer (Nova Biomedical, Waltham, Massachusetts, 02454 USA).

Whole blood samples were placed on ice packs immediately after and later transferred to a refrigerator upon arrival in the laboratory until analyzed. Time-sensitive analyses were performed within 12 hours. Packed cell volume and total solids were determined using high-speed centrifugation of blood-filled microhematocrit tubes.

### Statistics

Statistical analyses included comparisons of 18 biochemistry, blood gas, and hematology parameters between the three land iguana populations. For these comparative analyses we separated the two conspecific *C*. *subcristatus* population from North Seymour and South Plazas because they differed largely and significantly in body size and weight (see Results). Acknowledging small sample sizes and non-Gaussian distribution of most variables, we used nonparametric Kruskal-Wallis tests for the overall 3-group comparisons and the Wilcoxon ranked sum test for between group comparisons. The relationship between standard body size (SVL, snout-vent-length) and each biochemistry measurement was explored separately for each measurement using simple linear regression and Spearman rank correlation (r_s_). For regression/correlation analyses we also separated the two *C*. *subcristatus* conspecific island populations. The relationship between each biochemistry measurement and body condition, an indicator of an individual physiological status and general health [16], was also investigated through analogous statistical procedures as those used with standard body size. Body condition was assessed as a stardized body mass calculated as the ‘scaled mass index’ [16,17]. The R package (standardized) Major Axis Estimation and Testing Routines “smatr” 3.4–8 [18] was used for computing the scaling exponent using standardized major axis (SMA) regression. Haematocrit values from iSTAT and manual reads were compared separately for each species using paired comparisons Student t-tests. The potential effect of handling time on each blood biochemistry variable measured was assessed through nonparametric Spearman rank correlations analyses with Bonferroni correction. The effect of ectoparasite load (ticks) on PCV, total solids, hemoglobin (Hb) and body condition, on the other hand, was investigated by comparing animals containing at least one parasite (range one to four) with those free of parasites. For *C*. *subcristatus* from North Seymour, where number of ticks were recorded, we also performed a Spearman rank correlation between the number of ticks and PCV, total solids, hemoglobin (Hb) and body condition. All statistical analyses were conducted in R version 3.5.2 [19] and significance was set to an α-level of 0.05. The hybrid results were not included in the statistical analysis.

## Results

### Iguana health and morphometrics

**Fig 1:**
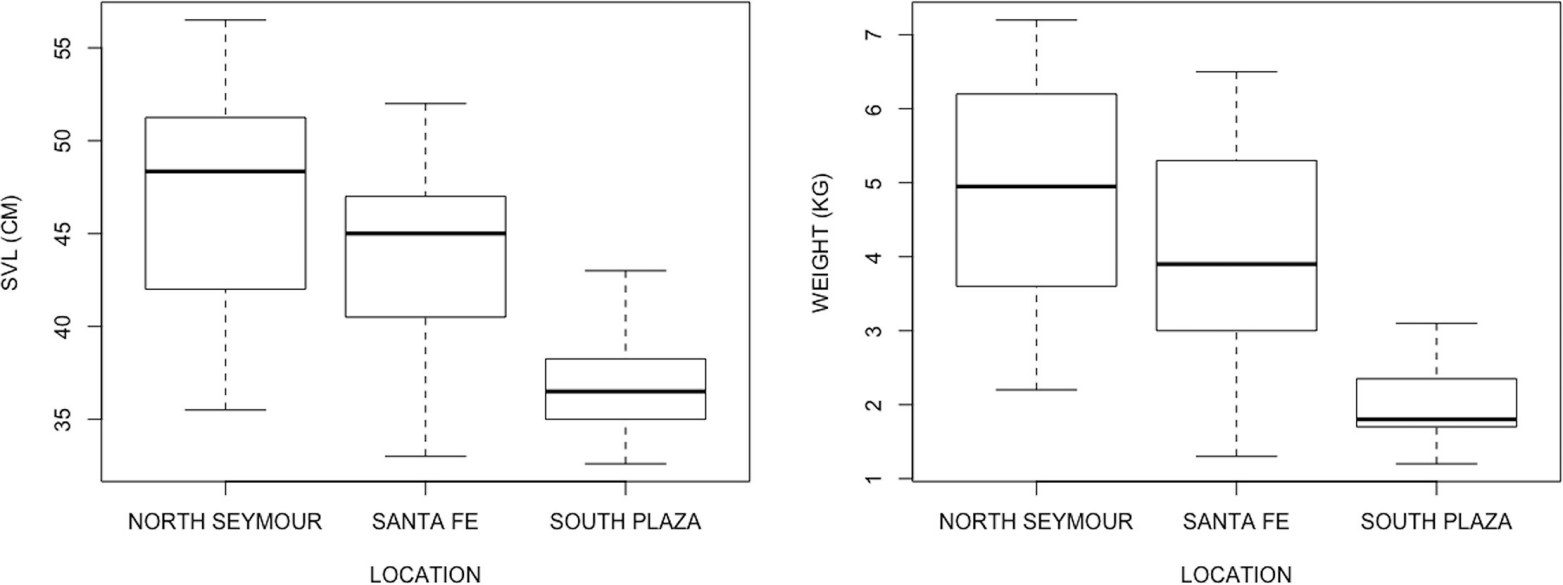
The interspecific population comparison among land iguanas from the three studied islands showed large and highly significant differences in body size (SVL: Kruskal Wallis Test: *X*^*2*^ = 17.484, df = 2, p< 0.001) and weight (Kruskal Wallis Test: *X*^*2*^ = 21.919, df = 2, p-value < 0.001).

Table 1 contains all of the morphometrics and health parameter data. *C*. *subcristatus* population from North Seymour was significantly larger (SVL, Wilcoxon rank sum test with continuity correction, W = 199.5, p< 0.001) and heavier (W = 213.5, p<0.001) than *C*. *subcristatus* from South Plazas. An interspecific three-population comparison rendered similar results with *C*. *pallidus* having an intermediate size and weight relative to the two conspecific *C*. *subcristatus* population (Fig 1).

**Table 1:** Mean, standard deviation, and range of morphometric and vital health parameters for wild Galápagos land iguanas with data included on marine iguanas [7].

	*C*. *subcristatus (North Seymour)*	*C*. *subcristatus*	*C*. *pallidus*	*A*. *cristatus*	*Hybrid*
	(n = 20)	*(South Plazas)*	(n = 21)	(n = 35)	(n = 1)
	Mean (Std. Dev.)	(n = 10)	Mean (Std. Dev.)	Mean (Std. Dev.)	Mean (Std. Dev.)
	Median	Mean (Std. Dev.)	Median	Median	Median
		Median			
Weight (Kg)	4.92(1.57)	1.97(0.59)	3.95(1.4)	4.9	2.7
	(2.2–7.2)	(1.2–3.1)	(2.00–6.5)	(1.5–8.3)	
	4.95	1.8	3.9		
Body Condition	3.89(0.87)	3.53(0.92)	3.68(0.76)	-----	Included with
	(2.6–6.09)	(2.55–5.4)	2.11–5.04)		South Plazas
	3.8	3.27	3.74		
SVL(cm)	46.75(5.92)	37.23 (3.52)	44.2(4.9)	40.5	36.5
	(35.5–56.5)	(32.6–43)	(33–52)	(28.5–51)	
	48.35	37	45		
TL (cm)	98.01 (12.02)	75.67 (6.59)	91.6 (7.8)	98.1	86.5
	(77.6–119)	(69.3–88)	(76–105.8)	(72.5–121)	
	99.6	74.5	91.1		
Girth (cm)	40.17 (5.54)	29.42 (3.33)	37.9 (6.3)	---------------	32.4
	(29.4–50)	(25–35)	(27–49)		
	40.8	28.5	37.5		
Heart Rate (bpm)	56.9 (11.25)	70 (9.29)	79.4 (14.2)	73	80
	(36–80)	(56–84)	(52–110)	(30–108)	
	58	68	80		
Respiratory Rate	19.2 (6.03)	33.6 (12.39)	23(9.7)	14	20
(bpm)	(12–36)	(24–56)	(8–50)	(8–22)	
	20	28	24		
Body Temperature	29.11 (3.92)	33.53 (1.53)	32.5 (2.7)	31.2	32
(^0^C)	(23.5–36.7)	(31.2–35.7)	(24.7–35.2)	(26.8–35.4)	
	29.7	33.4	33.1		

In *C*. *pallidus*, heart rate (r_s_ = 0.602, p<0.001) and respiratory rate (r_s_ = 0.519, p = 0.017) were largely dependent on body temperature. Similar strong correlations between heart rate (r_s_ = 0.724, p<0.008) and respiratory rate (r_s_ = 0.728033, p = 0.017) with body temperature, were found in *C*. *subcristatus* from South Plaza. However, in *C*. *subcristatus* from North Seymour only heart rate was significantly correlated with body temperature (r_s_ = 0.8761172, p<0.001).

All animals sampled were deemed clinically healthy based upon physical examination. Nine of the *C*. *subcristatus* (all from North Seymour) and seven of the *C*. *pallidus* were observed with ticks and one *C*. *pallidus* had small red mites. Ticks were collected in 70% ethanol but remain in the Galápagos for identification later. Sixteen of the *C*. *subcristatus* already possessed microchips at the time of capture as did two of the *C*. *pallidus*. If an animal did not have a microchip one was placed intramuscularly in the left femoral area.

### Blood analysis

Table 2 summarizes the biochemistry, blood gas, and hematology results for the 52 iguanas. Overall, the three populations differed significantly in 14 of the 18 biochemistry, blood gas, and hematology measurements (hearth rate, body temperature, lactate, respiration, BEecf, HCO3, TCO2, pHi, pHa, pCO2, iCa, glucose, HctPCV, Hb; Kruskal-Wallis, all p-values < 0.05). *C*. *pallidus* (Santa Fe island) differed from *C*. *subcristatus* of South Plaza in only two measurements (HctPCV, Hb; Wilcoxon rank sum test, all p-values < 0.05) while from *C*. *subcristatus* from North Seymour differed on 5 measurements (body temperature, respiration, pCO2, HctPCV, Hb; Wilcoxon test, all p-values < 0.05). The two *C*. *subcristatus* conspecific populations from North Seymour and South Plazas islands differed the most, with significance in eight of the 18 biochemistry, blood gas, and hematology measurements (heart rate, body temperature, respiration, BEecf, HCO3, TCO2, K, and iCa; Wilcoxon test, all p-values < 0.05).

**Table 2:** Mean, standard deviation, and range for blood gas and blood biochemical values for wild Galápagos land iguanas with data included on marine iguanas [7].

	*C*. *subcristatus (North Seymour)*	*C*. *subcristatus*	*C*. *pallidus*	*A*. *cristatus*	*Hybrid*
	(n = 20)	*(South Plazas)*	(n = 21)	(n = 35)	(n = 1)
	Mean (Std. Dev.)	(n = 10)	Mean (Std. Dev.)	Mean (Std. Dev.)	Mean (Std. Dev.)
	Median	Mean (Std. Dev.)	Median	Median	Median
		Median			
Blood sampling interval (min)	11.35 (3.25)	11.7 (4.16)	3.95 (1.4)	------------	19
	(7–19)	(7–18)	(2.00–6.5)		
	12	11	3.9		
BEecf	-11.2 (7.26)	-4.4 (8.17)	-11.7 (5.5)	------------	0
	(-24 to 7)	(-19 to 8)	(-24 to -2)		
	-12	2.5	-11		
HCO_3_^-^(mmol/L)	15.83 (4.88)	21.4 (6.7)	17.2 (4.5)	23.9 (4.7)	22.9
	(9.4–31)	(11.5–29.6)	(8.6–25.3)	(15.9–36.8)	
	15.15	22.9	16.8		
TCO_2_ (mmHg)	16.85 (4.83)	22.8 (6.96)	18.7 (4.8)	------------	24
	(11–32)	(13–31)	(10–27)		
	16.5	24	18		
sO2 (%)	93 (6.28)	92.6 (5.42)	86.4 (12.5)	80 (20)	99
	(82–100)	(82–99)	(52–97)	(35–99)	
	94.5	94.5	92		
pH_37_°_C_	7.26 (0.19)	7.33 (0.17)	7.15 (0.14)	_______	7.509
	(6.86–7.51)	(7.02–7.59)	(6.95–7.44)		
	7.29	7.32	7.15		
pH_Temp. corrected_	7.37 (0.23)	7.38 (0.17)	7.21 (0.14)	7.365 (.15)	7.586
	(6.89–7.66)	(7.06–7.63)	(6.97–7.50)	(6.96–7.65)	
	7.41	7.35	7.2		
pCO_2_ (mmHg)	25.48 (9.69)	35.25 (13.77)	41.2 (12.8)	41 (15)	23.1
	(10.8–46.9)	(26.72–43.78)	(18.9–61.6)	(19–90)	
	23.75	32.85	43		
pO_2_ (mmHg	63.7 (29.25)	65.3 (18,7)	60.5 (19.4)	45 (18)	83
	(28–123)	(40–102)	(29–98)	(22–86)	
	56.5	65	59		
Na (mmol/L)	155.1 (3.06)	151.9 (4.65)	153.7 (3.5)	178 (3)	168
	(149–160)	(145–159)	(145–157)	(169–180)	
	155.5	152	155		
K (mmol/L)	3.57 (0.37)	3.3 (0.41)	3.73 (1.05)*	4.3 (0.5)	3.2
	(2.6–4.1)	(2.8–4.2)	(<2–6.6)	(3.3–5.1)	
	3.65	3.3	3.9		
iCa (mmol/L	1.46 (0.1)	1.36 (0.09)	1.47 (0.17)	1.51 (.1)	1.33
	(1.21–1.59)	(1.16–1.45)	(0.94–1.67)	(1.2–1.68)	
	1.49	1.37	1.49		
Glu (mmol/L)	126.85 (10.62)	125.6 (9.24)	134.8 (12.4)	114 (12)	109
	(110–153)	(110–137)	(106–155)	(94–140)	
	125.5	126.5	135		
Lactate (mmol/L	6.78 (3.82)	6.21(2.51)	9.36 (3.13)	6.63 (4.01)	4.5
	(1.1–15.5)	(3.5–9.8)	(5.2–15)	(1.67–19.43)	
	5.85	5.6	8		
Hct_iSTAT_ (%)	21.05 (2.01)	20.7 (2.91)	28.95 (5.41)	27.05 (5.67)	28
	(18–26)	(17–26)	(16–37)	(17–37)	
	21	20.5	30		
PCV (%)	28.6 (2.23)	28.1 (3.870	39.7 (6.1)	37.96 (4.61)	36
	(24–31)	(24–36)	(31–52)	(28.3–44.9)	
	28	26.5	39		
TS (g/L)	51.5 (5.5)	48.1 (6.9)	72.4 (19.9)**	------------	46
	(42–66)	(42–64)	(50->120)		
	50	45	66		
Hb (g/L)	71.5 (6.8)	70.4 (9.7)	98.3 (18.5)	92 (19)	95
	(61–88)	(64.3–76.4)	(54–126)	(58–126)	
	71	70	102		

*The <2 value was given a value of 2.0 for the statistical analysis.

**The >12 value was given a value of 12 for the statistical analysis

The hematocrit figures generated by the iSTAT were significantly lower than the manually determined values in both species (*C*. *subcristatus*: t = 12.859, df = 30, p < 0.001; *C*. *pallidus*: t = -10.082, df = 20, p < 0.001) (Table 1).

**Fig 2:**
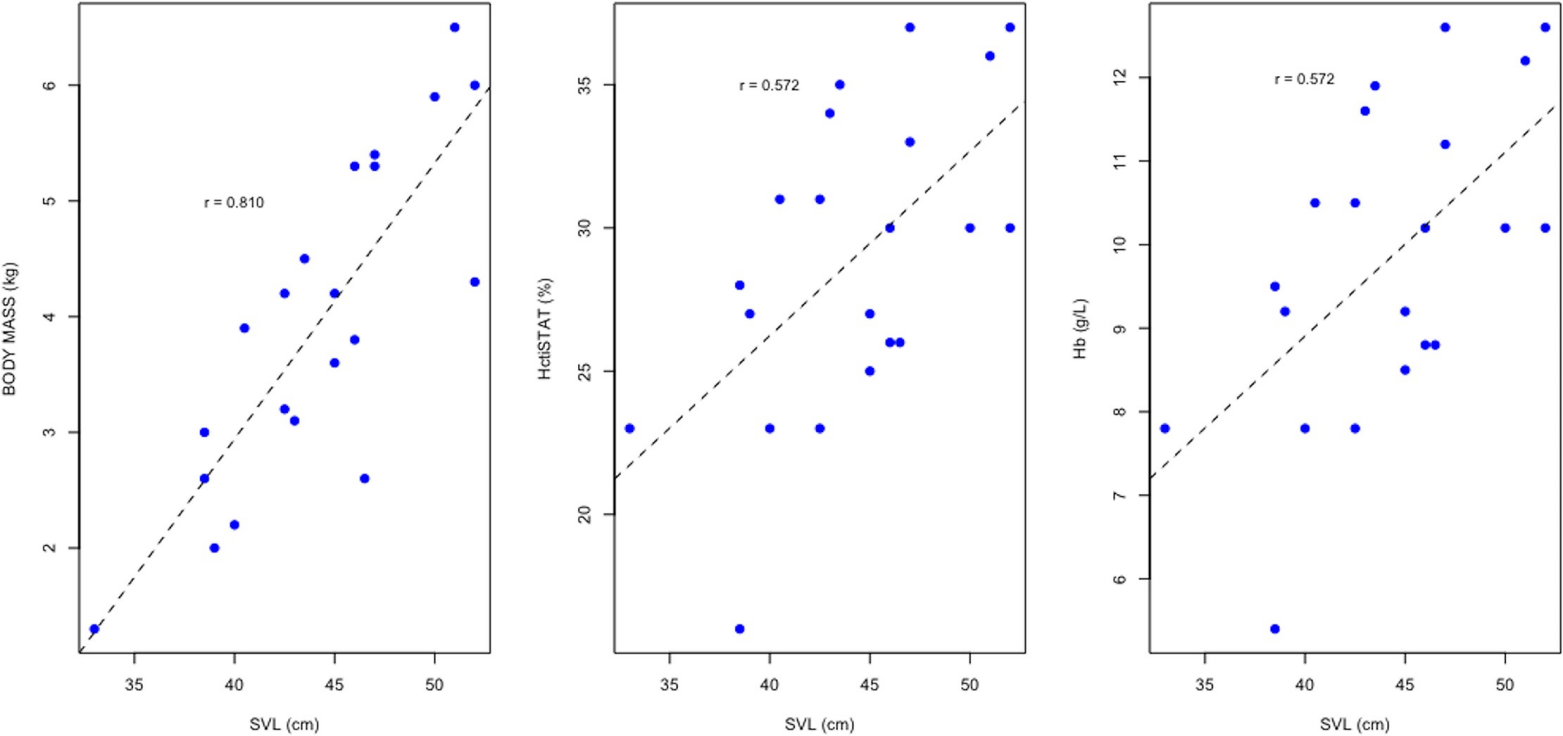
Linear regression and correlations of iSTAT hematocrit and hemoglobin with *C*. *pallidus* body size (SVL = Snout vent length). Linear regression models: BODY MASS (KG) = -6.60088 + 0.23858 (S_E_ = 0.03744)*SVL (t = 6.372, p = 4.11e-06); HctiSTAT (%) = 0.4580 + 0.6445 (S_E_ = 0.2034)*SVL (t = 3.168, p = 0.00507); Hb (g/L) = 0.10100 + 0.22012 (S_E_ = 0.06957)*SVL (t = . 3.164. , p = 0.00511). S_E_ refers to standard error.

Land iguanas of the three populations did not differ significantly in body condition (Kruskal-Wallis = 1.977, df = 2, p = 0.372). The standard body length (SVL) of *C*. *pallidus* was positively correlated with body mass (r_s_ = 0.810, p<0.001) and with only two blood measurements (HctPCV: r_s_ = 0.572, p < 0.001; Hb: r_s_ = 0.572, p < 0.001); see Fig 2. Likewise, in *C*. *subcristatus* from North Seymour only respiration rate correlated with standard body length (r_s_ = -0.52818, p = 0.039) and no correlations were found among *C*. *subcristatus* from South Plaza. Except for calcium (r_s_ = -0.375285, p = 0.049) in *C*. *pallidus*, blood biochemistry measurements did not relate to body condition in any land iguana species or population.

No differences in handling time were found among the three land iguana populations (Kruskal-Wallis = 0.49755, df = 2, p = 0.780), and handling time did not correlate with any health and blood biochemistry measured variables in any of the three land iguana populations (p>0.05 for all cases).

Among *C*. *pallidus* 33% (n = 21) had ectoparasites (ticks). Ticks were not found on *C*. *subcristatus* from South Plazas, but on North Seymour, 45% (n = 20) of the inspected iguanas had ticks, and the number of ticks among parasitized individuals ranged from one to four (median = two). The comparison between ectoparasitized and non-parasitized individuals showed no differences in any of the four variables both in *C*. *pallidus* (p≥0.08 for all cases) and in *C*. *subcristatus* (p≥0.07 for all cases). However, among ectoparasitized *C*. *subcristatus* we found a slight negative correlation between the number of ticks and PVC (r_s_ = -0.684, p = 0.042), hemoglobin (r_s_ = -0.684, p = 0.042), and body condition (r_s_ = -0.719, p = 0.029).

## Discussion

For proper health assessment veterinary clinicians desire species-specific baseline values from healthy individuals for parameters that can be easily and inexpensively measured with commercially available equipment. In ectotherms like reptiles, species specific data is especially important, due to diverse environmental conditions and varied habitats. Our study provides the first blood gas, biochemistry, and hematology measures in Galápagos land iguanas. Although the relatively small sample size (*C*. *subcristatus*, n = 30; *C*. *pallidus*, n = 21; *C*. *subcristatus X Amblyrhynchus cristatus* hybrid, n = 1) precludes the calculation of reference intervals [5], these results provide a useful preliminary baseline for veterinarians and other researchers. All iguanas we examined in this study were judged to be alert and clinically healthy; their blood parameters support this assessment.

When using point-of-care analyzers like the iSTAT it is important to be aware of their limitations. Several papers have examined this topic and have found that some blood gas and hematocrit values are not always accurate or reliable with certain non-mammalian species. In a study with the bar-headed goose (*Anser indicus*) it was determined that the iSTAT did not produce valid sO_2_ or hemoglobin values [20]. In rainbow trout (*Onchyrhynchus mykiss*) a strong validation study found that results varied with temperature and that only pH was a uniformly reliable value [21]. A similar study in sandbar sharks (*Carcharhinus plumbeus*) determined that the i-STAT is not a reliable device for accurately measuring blood gases in this species [22].

Most of the blood values we recorded for the two species of land iguanas and the hybrid were like those reported previously for other iguanids [23, 8, 12, 13, 6, 10]. For instance, the average PCV in green iguanas, *Iguana iguana*, [13] was 36.7%, very close to *C*. *pallidus* (39.7%), and nearly within the standard deviation range of the *C*. *subcristatus* mean (28.2%). The basilisk lizard (*Basiliscus plumifrons*) has a PCV (31.4%) much closer to *C*. *subcristatus* [8]. The blood sodium levels for green iguanas and basilisk lizards are 160 and 153.5 respectively [13, 8] while the sodium levels for *C*. *subcristatus* and *C*. *pallidus* are respectively 154 and 153.7. One indicator of health and low stress is blood glucose. It’s interesting that basilisk lizards had a fairly high level (203 mg/dL) while *C*. *subcristatus* and *C*. *pallidus* had much lower, more expected levels (126 and 135 respectively). There certainly might be a dietary component involved, but, the basilisk lizards were captured, held overnight in cloth bags, and sampled 12 hours later [8]. Thus, it is possible stress played a role in the glucose levels for species. Captive green iguanas had lower, more reasonable numbers, with mean glucose levels of 166 mg/dL for males and 180 mg/dL for females [13], and wild Allen Cays rock iguanas (*Cyclura cychlura inornata*) had a mean glucose of 189 mg/dL [9]. On the other hand, both captive and wild Cuban iguanas (*Cyclura nubila*) had much higher mean glucose levels, with 254 and 230 mg/dL respectively [23]. The authors attributed the higher glucose in captive animals with more frequent and higher quality meals.

In Table 1 we compare the results for the land iguanas with previously published data on the marine iguana [7]. Based on both morphological and DNA data we know that marine iguanas and land iguanas shared a common ancestor that might have lived about 4 million years ago [24, 25], when most, if not all, currently existing islands had not emerged yet [26, 27]. Despite of their old origin, *A*. *cristatus* and *C*. *subcristatus* can still hybridize on the island of South Plaza, generating a viable, yet most likely sterile F1 hybrid [28]. Despite being phylogenetically closely related, marine and land iguanas exhibit blood values that differ in many respects.

While smaller iguanas tend to have faster heart rates [29, 7] this was not the case with the land iguanas in our study. The two *Conolophus* species were not significantly different in weight, but, *C*. *pallidus* had significantly higher heart rates (mean of 79 compared to a mean of 61 for *C*. *subcristatus*). The larger marine iguanas had a mean heart rate of 73, between the two *Conolophus* species heart rates [7]. Respiratory rates were nearly identical between the two *Conolophus* but much faster than their marine counterpart. This difference is significant and likely results from the marine iguana’s physiological adaptation to breath holding.

The blood gas values and pH were fairly consistent between the three species, but the sodium levels were much lower than for marine iguanas [7], and nearly identical, between the land iguanas. This is most certainly due to the feeding and habitat difference between the terrestrial and marine iguanas. The marine iguanas also had higher potassium levels. The calcium values were comparable between all three species. The two species of land iguana had comparable blood glucose levels that significantly exceeded the value for the marine iguanas. This could also be related to nutrition and activity (with marine iguanas being much more active, thus requiring more energy, when feeding), or time of sampling after the last feeding.

The lactate levels were highest for *C*. *pallidus* but not significantly so. Along with having the lowest mean and lowest individual lactate level, *C*. *subcristatus* also displayed significantly lower PCV’s, total solids, and hemoglobin concentrations. We do not have an explanation for these lower values except that they are likely related.

Blood pH levels and subsequently lactate concentrations can increase rapidly due to excitement and activity in reptiles [7, 30]. Thus, handling of the animals was kept to a minimum to avoid affecting the measured blood chemistry results. The mean time to blood sampling from capture was 11.5 and 3.95 minutes respectively for *C*. *subcristatus* and *C*. *pallidus*. We don’t think sampling interval played a significant role in the results.

One final health consideration is the effect ectoparasites (in this case ticks) have on land iguana health. We examined four variables that might be affected by a blood-feeding ectoparasite; PCV, TS, Hb, and body condition. The comparison between ectoparasitized and non-parasitized individuals showed no differences in any of the four variables in *C*. *pallidus* and *C*. *subcristatus*. However, among ectoparasitized *C*. *subcristatus*, we found a slight negative correlation between the number of ticks and PVC, hemoglobin, and body condition. The impact of ectoparasites on these populations warrants further study.

In summary, our study represents an important step toward determining the normal range of values for blood gas, biochemistry, and other health parameters of Galápagos land iguanas. The animals we sampled all appeared clinically healthy. Regardless of the animal’s size and weight, nearly all blood parameters were consistent and similar, a good indicator of overall health. However, a validation study for point-of-care analyzers and iguanids is warranted and needed.

Galápagos land iguanas are protected vulnerable species with importance in the wildlife biology and conservation research community, health assessments are critical from the standpoint of wildlife protection and management. These results add to a growing database of knowledge about wildlife health management of Galápagos reptiles.

## Supporting information

S1 File “Biological Data & Samples”. (XLSX)

S2 File “Morphometrics & iSTAT Values”. (XLSX)
